# Synchronous multifocal osteosarcoma with hypocalcemia

**DOI:** 10.3402/ljm.v8i0.20359

**Published:** 2013-02-28

**Authors:** Li-Feng Qin, Han Fang, Li-Hua Qin, Xiao-ning Guo, Dan Peng

**Affiliations:** 1 Department of Orthopedics, the Second Xiang-Ya Hospital Central South University, Changsha, Hunan, China; 2 Department of Cardiovascular Disease, Xiang-Ya Hospital Central South University, Changsha, Hunan, China; 3 Department of gynecology, Nursing School, Hunan University of Chinese Medicine, Changsha, Hunan, China; 4 Department of Orthopedics, the Second Xiang-Ya Hospital Central South University, Changsha, Hunan, China; 5 Department of Orthopedics, the Second Xiang-Ya Hospital Central South University, Changsha, Hunan, China

To the Editor

Multifocal osteosarcoma (MOS) is a rare variant of osteosarcoma that is defined by two or more tumors at any site of the peripheral or axial skeleton (1). MOS can be categorized into two types: synchronous (multiple lesions that appear to develop within 6 months) and metachronous (new lesions that develop over a time interval longer than 6 months) (2). Synchronous MOS first appears during childhood or early adolescence and has been reported to account for approximately 0.4–4.2% of all osteosarcoma cases (3, 4), which occurs in nearly symmetrical and multiple locations in the metaphyseal of long bones (1).

On February 11, 2011, a 14-year-old male initially presented with pain in the right thigh muscle without trauma and was subsequently found to have purely osteosclerotic lesions of the skull, vertebrae, ribs, bilateral and nearly symmetric proximal humerus, pelvis, ilium, sacrum, proximal and distal femurs, and proximal tibiae (Figs. 1 and 2). The lesions were particularly evident in the right distal femur (Fig. 3). Laboratory tests revealed hypocalcaemia, elevated parathyroid hormone, alkaline phosphatase (ALP), and lactic dehydrogenase (LDH) (Table 1). A lumbar intervertebral disc protrusion was also found by computed tomography (CT).

**Fig. 1:**
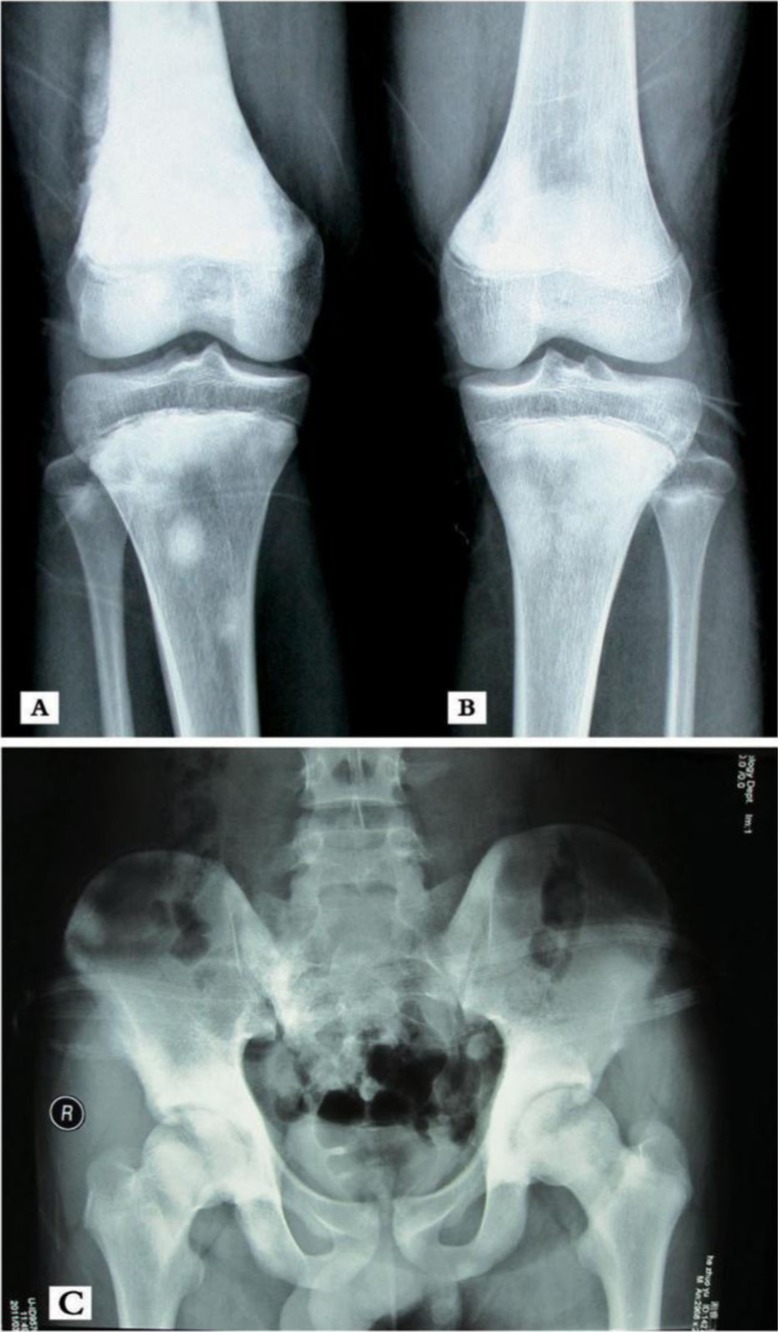
X-ray showing pure, patchy osteosclerotic lesions of (A) distal femurs, (B) proximal tibiae, and (C) hip bones, ilium, sacrum, and proximal femurs. Bony destruction is likely evident in the right distal femur and appears to be surrounded by a patchy periosteum reaction.

**Fig. 2:**
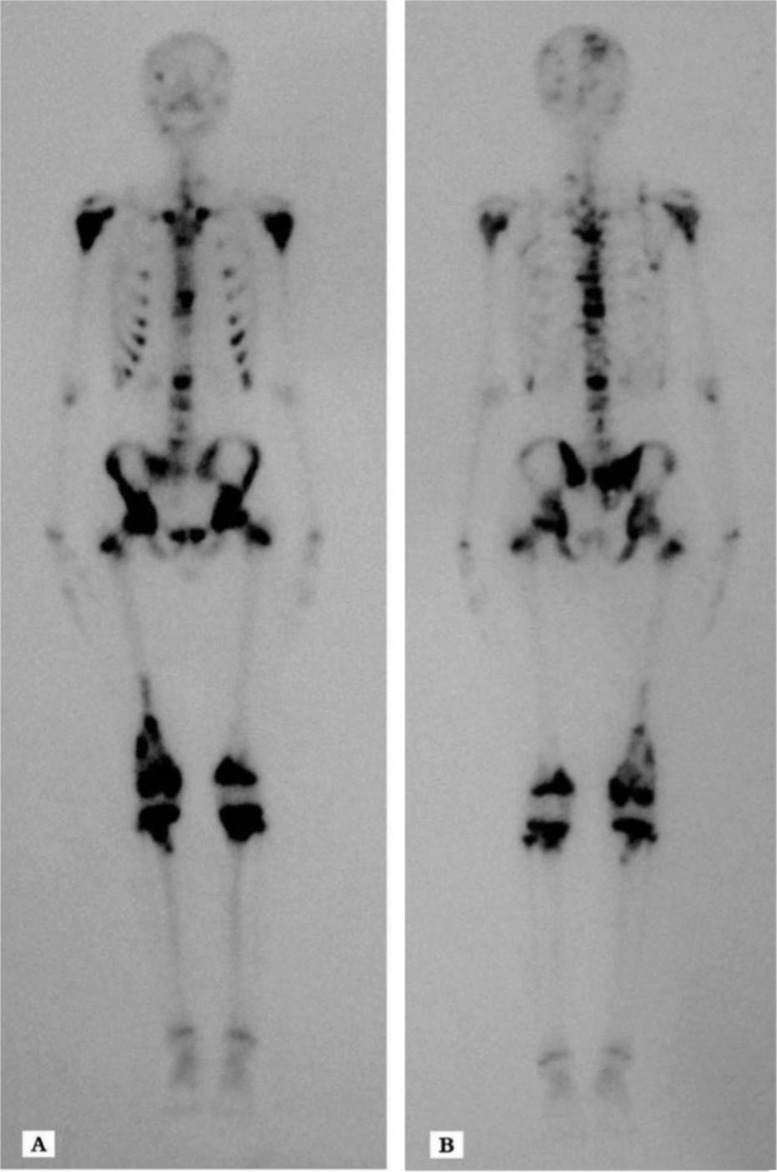
An (A) anterior and (B) posterior bone scan showing multiple foci in the skull, vertebrae, ribs, bilateral and nearly symmetric proximal humerus, pelvis, ilium, sacrum, proximal and distal femurs, and proximal tibiae with abnormal bone uptake.

**Fig. 3:**
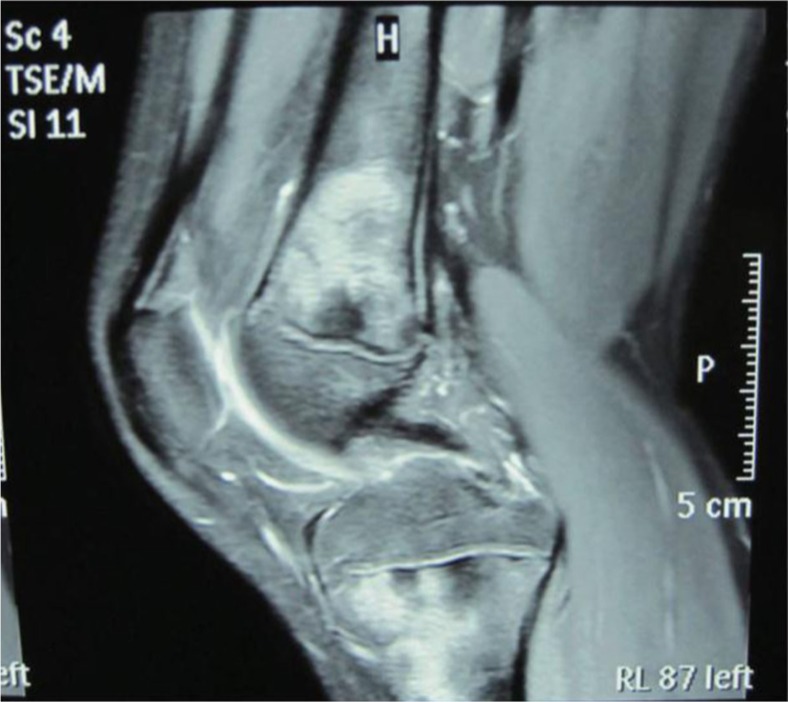
MRI of the left knee showing marked flakes and nodular long signals on T2-weighted MRI in the distal femur, proximal tibia, and even the osteoephysis.

**Table 1:** Biochemical parameters before and after chemotherapy.

Date (2011)	Parathyroid hormone	24-h urinary calcium	24-h urinary phosphorus	Serum calcium	Serum phosphorus	Alkaline phosphatase
Mar 3	15.39 and 19.94	1.12	59.25	2.17	1.64	1874.4
Mar 5	16.43 and 20.95	0.75	26.34	2.17	1.71	1974.3
Mar 7				1.95	1.54	
Mar 18	44.97 and 42.91			1.73	1.18	
Mar 31				1.81	1.35	2208.39
Apr 7				1.48	1.26	1608.5
Apr 9				1.52	1.19	
Apr 11				1.89	1.19	
Apr 17				1.95	1.49	1561.8
May 2				1.93	1.48	1519.5
May 5				1.69	1.33	1334.7
May 9				2.18	1.46	1222.8
May 13				2.05	1.54	954.9

Parathyroid hormone (PTH) (normal range: 1.48–7.63 pmol/L); 24-h urinary calcium (normal range: 5.0–6.25 mmol/day); serum calcium (normal range for children: 2.25–3.0 mmol/l, adult: 2.1–2.75 mmol/L); serum phosphorus (normal range for children: 1.29–1.94 mmol/L, adult: 0.97–1.60 mmol/L); alkaline phosphatase (ALP) (normal range: 30.0–110.0 U/L).

A blood gas analysis, repeated twice, showed a mild generation of acid; however, other laboratory values were all within normal ranges. The detected levels of serum phosphorus, 24 h urinary calcium and phosphorus, and parathyroid hormone are shown in Table 1. The level of 25-hydroxy vitamin D was 9 pg/mL (normal range: 10–55 pg/mL), while the level of 1, 25-dihydroxy vitamin D was 130 pg/mL (normal range: 15–90 pg/mL). The bone mineral density (BMD) analysis was 0.5% lower than that typical of adolescents of a similar age. Analyses of thyroid, parathyroid, abdominal B ultrasonography, double-lower extremity arteriovenous color Doppler, and echocardiography were all normal.

The clinical presentation was initially controlled by Celebrex, and the differential diagnosis prior to biopsy included hyperparathyroidism, Paget's disease (teenager's type), osteopetrosis, melorheostosis (leri) (variant type), skeletal fluorosis, Erdheim-Chester disease, and osteosarcoma. Serum calcium levels continued to decrease after treating the patient with chewable vitamin D tablets (200 U/day), calcitriol soft capsules (0.75 µg/day), Celebrex (200 mg/day), and intravenous (i.v.) Aclasta (5 mg/day). Since the treatment regimen alleviated the muscle pain, the patient was voluntarily discharged on March 19, 2011, despite persistent hypocalcemia. After discharge, the patient gradually developed numbness and weakness of both lower limbs with subsequent dysuria. On March 30, 2011, he was admitted to the department of orthopedics at our hospital. Physical examination showed that the patient had a positive straight leg raise test in the lower extremities of both appendages, hypopselaphesia below the navel plane, and class III-V muscle strength in both lower limbs. Hypocalcemia was again identified by laboratory tests, while the hemoglobin, white blood cell count, platelet count, blood urea nitrogen, serum creatinine, aspartate aminotransferase (AST), C-reactive protein (CRP), erythrocyte sedimentation rate (ESR), and albumin were all within normal ranges. The magnetic resonance imaging (MRI) revealed a tumor compressing the fifth thoracic spinal cord. An x-ray of the lungs was normal.

An open bone biopsy was performed on the bilateral distal femora and proximal tibiae, and four foci were found that exhibited mid-grade osteoblastic osteosarcoma (Fig. 4). Based on the imaging, pathology findings, and clinical manifestation, a diagnosis of MOS was ultimately established (synchronous MOS). Following administration of neoadjuvant chemotherapy with a first cycle of methotrexate (MTX), pirarubicin (THP), and ifosfamide (IFO), the serum calcium level returned to normal, although the clinical symptoms continued to progress. A CT scan of the lung showed no signs of tumor metastases. In comparison to the previous MRI (Fig. 5B and C), the number of lesions had increased and the tumors in the spinal canal were amplified after the neoadjuvant chemotherapy (Fig. 5A). Due to the progression of clinical symptoms after chemotherapy, the patient's parents made the decision to discharge the patient from hospital. At the 4-month follow-up, which was conducted by telephone, we determined that the patient's symptoms had continued to deteriorate, and the patient died 15 months after diagnosis.

**Fig. 4:**
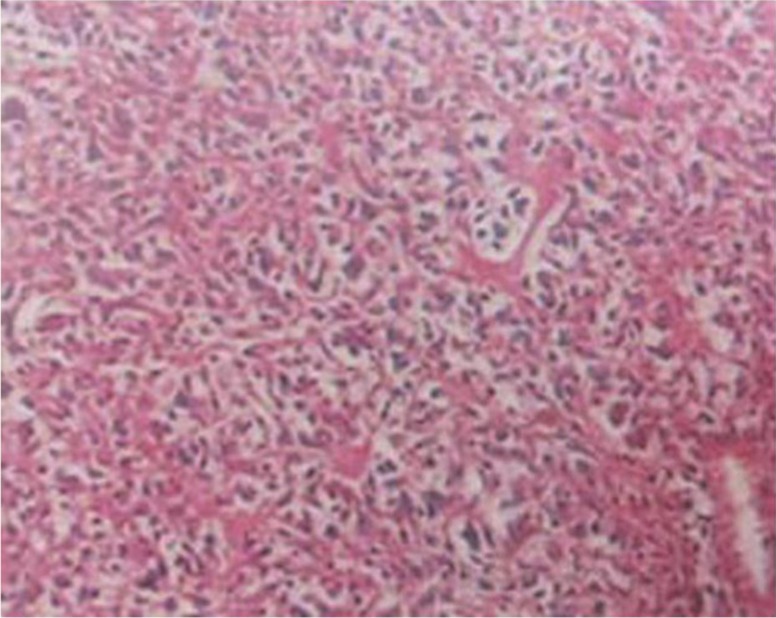
Hematoxylin and eosin (H&E) staining (magnification×100) indicates tumor cells producing osteoid.

**Fig. 5:**
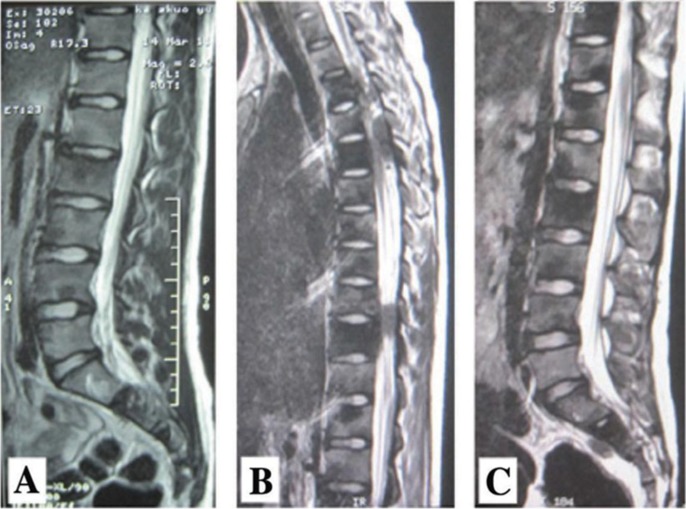
Thoracolumbar MRI (A) shows a short signal on T2-weighted MRI in the T2 to T11, L1 to L5, and sacral vertebra. (B, C) Flake low signal shadows are observed outside of the spinal cord and dura in the T4-T5 and T9-T10 spinal body plane. MRI taken after neoadjuvant chemotherapy (A) indicated that the number of lesions increased and the tumors in the spinal canal were amplified, as compared to that taken before treatment (B, C).

## Discussion

Synchronous MOS is a highly aggressive variant of osteosarcoma that presents as multiple bone lesions at the time of diagnosis and usually without pulmonary metastases. This patient exhibited sufficient urinary conservation of calcium and phosphorus, as indicated by the 24 h urinary calcium and phosphorus levels (Table 1). The rising level of serum parathyroid hormone (PTH) and 1, 25-dihydroxy vitamin D showed normal responses to prolonged hypocalcemia, and the patient was able to produce high levels of 1, 25-dihydroxy vitamin D despite low levels of 25-hydroxy vitamin D. The results of the BMD test showed a level 0.5% lower than that of adolescents of a similar age. Previous studies have indicated that possible explanations for low levels of 25-hydroxy vitamin D in Asian children may include increased bone formation, lower ultraviolet exposure, and insufficient dietary consumption (5, 6). It should be noted, however, that the serum calcium level was normal in this patient upon initial assessment, and hypocalcemia rapidly developed after patient care had commenced. The serum calcium did not reach a normal level until the patient had received a complete first cycle of neoadjuvant chemotherapy. In view of the elevated PTH and ALP levels, the severe and refractory hypocalcemia in this patient was most likely caused by rapid and continued deposition of calcium by the malignant osteoblasts into the tumor osteoid (7–9).

To date, approximately 100 cases of synchronous MOS have been reported in the literature. It remains unknown whether hypocalcaemia is associated with a poor prognosis of MOS. Hypocalcemia in patients with osteosarcoma is extremely rare, and only four cases of MOS with hypocalcemia have been reported in the literature to date (7–10). Of these cases, one MOS patient with non-symptomatic hypocalcemia responded to calcitriol but did not respond to i.v. and oral calcium supplementation (10), two OS patients’ hypocalcemia appeared during treatment (7, 8), and one MOS patient had obvious symptomatic hypocalcemia from the early phases of the disease (9). Hypocalcemia in our patient first appeared during assessment and failed to respond to i.v. and oral calcium and calcitriol supplementation. The serum calcium level only became normal after aggressive neoadjuvant chemotherapy (Table 1); however, the clinical symptoms continued to progress (Fig. 5). There is currently no standard of care for MOS patients, and it has been reported that all nine patients with MOS analyzed had died between 6–17 months after diagnosis (average 12 months) (11).

In summary, we have presented a rare case of synchronous MOS with hypocalcemia together with a current literature review. We conclude that it is still difficult to establish diagnosis standards for synchronous MOS, and targeted therapies should not be administered until sufficient biopsy and pathological findings are provided, since synchronous MOS has a similar presentation as metabolic bone diseases and significant clinical improvements from these therapies have not been achieved. Importantly, we suspect that hypocalcemia during MOS may be associated with the disease progression and poor prognosis. Further investigations are needed to explore these possibilities. Hypocalcaemia could be a manifestation of this condition and therefore the clinician should be aware of this possibility.

*Li-Feng Qin* Department of Orthopedics, the Second Xiang-Ya Hospital Central South University, Changsha Hunan, China*Han Fang* Department of Cardiovascular Disease, Xiang-Ya Hospital Central South University, Changsha Hunan, China*Li-Hua Qin* Department of gynecology, Nursing School Hunan University of Chinese Medicine, Changsha Hunan, China*Xiao-ning Guo* Department of Orthopedics, the Second Xiang-Ya Hospital Central South University, Changsha Hunan, China*Dan Peng* Department of Orthopedics, the Second Xiang-Ya Hospital Central South University, Changsha Hunan, China Emails: xyeypdyz@163.com; qinlifeng813@hotmail.com
